# The Institutional Worker

**Published:** 1912-01-06

**Authors:** 


### THE INSTITUTIONAL WORKER

**Being a Special Supplement to "The Hospital"**

### Editor's Notices.

**Contributions:**

Contributions should be written, or preferably typed, on one side of the paper only, and all articles sent in are accepted upon the distinct understanding that they are forwarded to The Hospital only.

The Editor cannot undertake to return MSS. not used, but when a stamped directed envelope is enclosed the MSS. may be returned if a special request is made.

Accepted articles and paragraphs of news will be paid for after publication at the scale rate.

**Address:**

To prevent delay all contributions and letters on editorial business must be addressed exclusively to the Editor, "The Hospital," 29 Southampton Street, Strand, London, W.C. It is important that this regulation shall be strictly observed.

**Correspondence:**

Correspondence on all subjects is invited. The name and address of correspondents must be given as a guarantee of good faith, but not necessarily for publication.

**Special Articles:**

Special articles are invited, and questions, inquiries, and paragraphs upon all matters relating to the work, administration, and management of general, special, mental, fever, cottage and convalescent hospitals, sanatoria, homes, institutions, societies and organisations for the treatment or care of the sick, injured and dependents of all classes. Every matter affecting the interests and welfare of the staff of all grades working in these institutions will receive special consideration.

**Administrative Medicine, Research and Items of News:**

Special terms will be paid for approved articles on subjects in this wide field by experts who have made some section of it their special study and interest. We wish to give prominence to every movement tending to advance accuracy of diagnosis, efficiency in treatment, and the development of modern methods to eradicate disease and promote the welfare of the suffering.

**A Bureau of Information:**

We invite inquiries and applications for information and help in providing for that numerous class of sufferers who require special care or house-room which they cannot obtain in their own homes or secure for themselves. We cannot, however, prescribe or recommend practitioners.

**Books for Review:**

Publishers are particularly requested to send advance proofs of any new books of importance whenever possible, as the Editor has made arrangements to publish immediate reviews on a new plan.

**Photographs, Plans, Blocks, and Illustrations:**

It is requested that wherever possible MSS. may be accompanied by illustrations in any of the above forms. The name of the sender and of the article to which it belongs should be written on the back of each photograph, drawing, or block for purposes of identification.

**Local Papers:**

Newspapers containing reports or news paragraphs should be marked and addressed to the Sub-Editor.

**The Coupon System:**

A coupon will be found at the bottom of the third inside page of the cover each week. This coupon must be attached to every question or inquiry to which an answer is desired in The Hospital.

### Manager's Notices.

Letters relating to the publication, sale, and advertising departments of The Hospital must be addressed to the Manager, "The Hospital," The Hospital Building, 28 and 29 Southampton Street, Strand, London, W.C.

Subscriptions which may begin at any time, are payable in advance. Cheques and Post-Office orders, crossed London County and Westminster Bank, Covent Garden branch, should be made payable to the Manager of The Hospital and addressed to him at The Hospital Building, 28 and 29 Southampton Street, Strand, London, W.C.

**Advertisements:**

All advertisements for The Hospital must reach the Manager not later than Wednesday morning in each week. For scale of charges see pages v and 2.

**Classified Advertisements:**

All small advertisements will be classified, for this section of the paper is widely read and of special interest. We wish to encourage those wanting appointments who are attracted to institutional work, and therefore offer them the reduced rate of *twenty-one words and under for* 1s., and *for every ten and part of ten words beyond, 6d.*

### POINTS FOR INSTITUTION AUTHORITIES.

The economical management of an Institution depends very greatly upon purchasing supplies in the cheapest market.

It is often impossible locally to secure the highest quality at the lowest prices.

In consequence, a section will be reserved in this part of the paper for announcements of Institutions inviting Tenders for Contracts.

This will enable Authorities of Institutions to secure tenders for supplies from all over the country, and ensure purchases at the lowest possible prices consistent with quality.

The charge for Tenders is 6s. per inch, and for this small outlay many pounds may be saved on supplies of all descriptions.

Special Rates will be quoted for those institutions which agree to send all their vacancy, contract, and public notices to The Hospital each year, so as to make it a file of such announcements for ready reference.

### POINTS FOR TRADE ADVERTISERS.

The Hospital is the organ of Institutions, whether of a Voluntary Character or those under Local Government, Poor Law and Municipal Authorities.

It is read by all Executive Officials and those responsible for the Construction, Management, and Equipment of Public Establishments.

It is also the Journal of the worker engaged in the Medical, Health and Preventive services.

There is no medium more appropriate and effective in the promotion of business in these directions than The Hospital.

### POINTS FOR INSTITUTIONAL WORKERS.

A very large number of Appointments Vacant appear every week in the columns of The Hospital.

If you cannot find a position suited to your requirements, make your wishes known through our Situations Wanted Column.

### APPOINTMENTS VACANT.

### Poor-Law Vacancies.

### Appointments Vacant.

ASSISTANT FOSTER-MOTHER (£25), St. Albans Union. Mr. R. W. Brabant, C. to G., St. Albans. Jan. 11.

ASSISTANT MATRON (£17), Rye Union. Mr. T. J. Smith, Flushing House, Rye. Jan. 19.

ASSISTANT NURSE (£30), Bury Union. J. Isherwood, C. to G., Union Offices, Bury. Jan. 11.

ASSISTANT NURSE (£25), Bedford Union. Mr. W. Payne, C. to G., Bedford. Jan. 8.

ASSISTANT NURSE (£25), Evesham Union. E. H. Wadams, C. to G., Union Offices, Evesham. Jan. 6.

ASSISTANT NURSE (£25), Windsor Union. P. Lovegrove, C. to G., 3 Park Street, Windsor. Jan. 13.

ASSISTANT NURSE (£25), Hartley Wintney Union. W. H. Wright, C. to G., Odiham, Hants. Jan. 11.

ASSISTANT NURSES (£26), Huddersfield Union. Mr. E. A. Rigby, C. to G., Huddersfield. Jan. 11.

BOYS' ATTENDANT (£24), Ipswich Parish. Mr. L. W. Greenhalgh, C. to G., Ipswich. Jan. 9.

CERTIFICATED MIDWIFE (£35), St. Marylebone Guardians. Matron, Northumberland Street Workhouse, Baker Street, W.

CHARGE AND MIDWIFERY NURSE (£32), Northampton Union. W. Fawkes, C. to G., 4 Derngate, Northampton. Jan. 10.

CHARGE NURSE (£30), Richmond Union. Mr. P. Umney, C. to G., Parkshot, Richmond, Surrey. Jan. 10.

CHARGE NURSES (£30), Mutford and Lothingland Union. F. Peskett, 148 London Road, Lowestoft. Jan. 12.

CHILDREN'S ATTENDANT (£24), Newport Union. Mr. A. H. Rees, C. to G., Newport, Mon. Jan. 10.
CLERK (15s.), Shoreditch Guardians. Mr. R. Clay, 213 Kingsland Road, N.E. Jan. 13.
FEMALE ATTENDANT (£27), Monyhull Colony for Epileptics, King's Heath, Birmingham. Matron.
FEMALE ASSISTANT EPILEPTIC WARD ATTENDANT (£18), Holborn Union. Matron of the Workhouse, Mitcham, Surrey.
FOSTER-MOTHER (£25), Bishop Stortford Union. Mr. A. G. Gwynn, C. to G., Bishop Stortford. Jan. 6.
FOSTER-MOTHER (£24), Meriden Union. Mr. A. W. Liggins, C. to G., 11 Priory Street, Coventry. Jan. 11.
GIRLS' AND INFANTS' ATTENDANT (£25), Chesterfield Union. R. F. Hartwright, C. to G., Union Offices, Chesterfield. Jan. 13.
HEAD NURSE (£45), East Preston Union. Mr. A. Shelley, C. to G., Littlehampton. Jan. 13.
INDUSTRIAL TRAINER AND FOSTER-MOTHER (married couple), (£35 and £30), Kingston Union. Mr. C. W. Dash, C. to G., Kingston-on-Thames.
INFANTS' ATTENDANT (£25), Darlington Union. Mr. C. H. Leach, C. to G., Darlington. Jan. 6.
INFANTS' NURSE (£25), Bucklow Union. G. Leigh, C. to G., Union Offices, Knutsford. Jan. 22.
LABOUR MASTER (£27 10s.), Chesterfield Union. Mr. R. F. Hartwright, C. to G., Chesterfield. Jan. 6.
MALE LUNATIC ATTENDANT (£30), Parish of St. Giles, Camberwell. Mr. C. S. Stevens, C. to G., 29 Peckham Road, S.E. Jan. 11.
MALE LUNATIC ATTENDANT (£30), Parish of St. Marylebone. Master, Workhouse, Northumberland Street, W.
MALE NURSE (£32), Paddington. Mr. H. F. Aveling, C. to G., 313 Harrow Road, W. Jan. 10.
MALE NURSE FOR EPILEPTICS, ETC. (£30), Lambeth Parish. Master of the Workhouse, Lambeth.
NIGHT SISTER (£38), Sculcoates Union. J. H. Wild, C. to G., 12 Harley Street, Hull. Jan. 17.
NIGHT SISTER and ASSISTANT SUPERINTENDENT NURSE (£36), Newport (Mon.) Union. A. H. Rees, C. to G., Union Offices, Queen's Hill, Newport (Mon.). Jan. 10.
NURSE (£26), Chesterton Union. Mr. J. S. Symonds, 9 Bene't Street, Cambridge. Jan. 12.
NURSE (£30), Elham Union. E. Lovick, C. to G., 29 Bouverie Square, Folkestone. Jan. 17.
NURSES (£23), Greenwich Union. Mr. S. Saw, C. to G., East Greenwich.
NURSES (£30), Gateshead Union. G. Craighill, C. to G. Poor Law Union Offices, Gateshead. January 6.
NURSES (£28), Newton Abbot Union. Mr. F. Horner, C. to G., Newton Abbot. Jan. 9.
NURSES (2) (£31 11s.), East Preston Union. Mr. A. Shelley, C. to G., Littlehampton. Jan. 13.
NURSERY ATTENDANT (£30), Prestwich Union. Mr. E. W. Ogden, Union Offices, Cheetham Hill Road, Manchester. Jan. 8.
PROBATIONER AND NURSES (£10), Burnley Union Infirmary. Mr. J. S. Horn, Union Offices, Burnley.
PROBATIONER NURSE (£12), Isle of Wight Union. Mr. A. G. Harrison, 30 Pyle Street, Newport, I.W. Jan. 9.
PROBATIONER NURSE (£10), Hackney Union. Matron, Hackney Union Infirmary, Homerton.
SENIOR CHARGE NURSE (£32), Tonbridge Union. Mr. N. R. Stone, C. to G., 23 Church Road, Tonbridge. Jan. 11.
SISTER (£35), Medway Union. A. Reynolds Norman, C. to G., 22 High Street, Chatham. Jan. 16.
STAFF NURSE (£30), Kidderminster Union. Mr. F. E. Burcher, C. to G., Kidderminster. Jan. 8.
STAFF NURSE (£25), Winchester Union. Mr. F. Faithfull, C. to G., Winchester. Jan. 8.
STAFF NURSE (£24), Holborn Union. Matron of the Workhouse, 1A Shepherdess Walk, N.
STAFF NURSES (£24), Wandsworth Union. F. W. Piper, Union Offices, St. John's Hill, Wandsworth, S.W.
STEWARD'S CLERK (15s. weekly), Kensington Guardians. The Steward, Kensington Infirmary, Marloes Road, W.
SUPERINTENDENT NURSE (£38), Bedford Union. Mr. W. Payne, C. to G., Bedford Jan. 8.
SUPERINTENDENT OF FEMALE LUNATIC WARDS (£45), South Manchester Guardians. D. S. Bloomfield, C. to G., All Saints, Manchester. Jan. 16.
TEMPORARY RELIEVING OFFICER (£2), Brighton Parish. Mr. B. Burfield, C. to G., Brighton.
TRAINED NURSE (£30), Fylde Union. Mr. F. H. Brown, C. to G., Union Offices, Wesham, Kirkham. Jan. 9.
WARD SISTER (£30), Bermondsey Guardians. Matron of the Infirmary, Lower Road, Rotherhithe.

### Situations Vacant.

ASSISTANT COOK (FEMALE) (£20), Greenwich Union. Mr. S. Saw, C. to G., Greenwich. Jan. 13.
ASSISTANT COOK (£22), Bethnal Green Guardians. The Matron, Bethnal Green Infirmary, Cambridge Road, N.E.
BOYS' (MALE) COOK ATTENDANT (£30), Farnham, etc., District School. Superintendent, District Schools, Crondall, Hants.
COOK AND ASSISTANT TO MATRON (£30), Huddersfield Union. Mr. E. A. Rigby, C. to G., Huddersfield. Jan. 11.
GENERAL ASSISTANT (£20), Parish of Leicester. Mr. H. Mansfield, Poor-Law Offices, Leicester. Jan. 8.
KITCHENMAID (£20), Edmonton Union. Matron, Infirmary, 77 Bridport Road, Upper Edmonton.
KITCHENMAID (£14), Saffron Walden. Mr. J. A. S. Baily, C. to G., Saffron Walden. Jan. 8.
LAUNDRESS (£27 10s.) Thetford Union. Mr. J. Houchen, C. to G., Thetford.
LAUNDRESS (£25), Bishop Stortford Union. Mr. A. G. Gwynn, C. to G., Bishop Stortford. Jan. 6.
PORTER AND PORTRESS (£36-£26), Romford Union. Mr. C. Bloomfield, C. to G., Workhouse, Romford. Jan. 8.
SENIOR HOUSEMAID (£18), Edmonton Union. Matron, Infirmary, 77 Bridport Road, Upper Edmonton.
SORTER AND PACKER (£28), Prestwich Union. Mr. E. W. Ogden, Union Offices, Cheetham Hill Road, Manchester. Jan. 8.

### Institutional and Administrative Vacancies.

### Appointments Vacant.

LADY SUPERINTENDENT (£65), West Kent General Hospital, Maidstone. Secretary. Jan. 15.
MATRON (£80), Oldham Royal Infirmary. E. Lionel Blake, Superintendent-Secretary. Jan. 15. (See advt.)
MATRON (£63), Royal Albert Hospital, Devonport. Captain C. W. Dickinson, R.N., Secretary. Jan. 10.
SISTER (£30), Queen's Hospital, Birmingham. Apply, Matron. Jan. 7.
SISTER (£35), Electrical and X-Ray Department, Charing Cross Hospital. Matron. Jan. 12.
THEATRE SISTER (£32), Warrington Infirmary. Apply Matron.
THEATRE SISTER (£35), Oldham Royal Infirmary. E. Lionel Blake, Superintendent-Secretary. Jan. 15. (See advt.)
SISTER (£35), Oldham Royal Infirmary. E. Lionel Blake, Superintendent-Secretary. Jan. 15. (See advt.)

### Municipal Vacancies.

### Appointments Vacant.

ASSISTANT COUNTY ROAD SURVEYOR (£200), County of Worcester. Mr. L. Thornley, Shire Hall, Worcester. Jan. 10.
ASSISTANT INSPECTOR OF NUISANCES AND HEALTH VISITOR (£100), Borough of Stafford. Mr. R. Battle, Town Clerk, Borough Hall, Stafford. Jan. 8.
ASSISTANT SANITARY INSPECTOR (£100), Borough of Shrewsbury. Mr. H. C. Clarke, Town Clerk, Shrewsbury. Jan. 8.
BOROUGH SURVEYOR AND ENGINEER (£600), Southport T.C. Mr. J. E. Jarratt, Town Clerk, Southport. Jan. 15.
CHIEF CONSTABLE (£350), Lincoln T.C. Mr. W. T. Page, Town Clerk, Lincoln. Jan. 6.
CLERK (£52), Broadstairs and St. Peter's U.D.C. Mr. L. A. Skinner, Clerk to the Council, Broadstairs. Jan. 6.

